# Therapeutic efficacy of spirulina against ovalbumin and cigarette smoke‐induced asthma‐specific stress biomarkers in Sprague–Dawley rats

**DOI:** 10.1002/fsn3.3132

**Published:** 2022-11-14

**Authors:** Khadija Riaz, Masood Sadiq Butt, Mian Kamran Sharif, Muhammad Naeem Faisal

**Affiliations:** ^1^ National Institute of Food Science & Technology, Faculty of Food, Nutrition & Home Sciences University of Agriculture Faisalabad Pakistan; ^2^ Faculty of Veterinary Science, Institute of Physiology and Pharmacology University of Agriculture Faisalabad Pakistan

**Keywords:** asthma, bronchoalveolar fluid, cigarette smoke, Ovalbumin, spirulina, stress biomarkers

## Abstract

Due to the high prevalence of allergies and asthma, awareness about allergens and therapeutic use of functional foods and nutraceuticals have gained immense attention. Spirulina powder is being used as health‐boosting and antioxidant agent against several ailments owing to its unique nutritional profile. Considering its antioxidant role, the current study was focused on exploring therapeutic role of spirulina against stress biomarkers in asthmatic model. To assess the therapeutic efficacy of spirulina against allergic asthma‐specific oxidative stress biomarkers, a model feed trial was conducted and rats were divided into four groups (*n* = 10). G_0–I_ (negative control), G_0–II_ (positive control), whereas G_I_ (spirulina) and G_2_ (salbutamol) served as treatment groups. Salbutamol is a chemical compound which is used in several antiallergic medicines because it works as bronchodilator. G_2_ group was given salbutamol for comparison of results. For asthma induction, rats were given intraperitoneal injection of ovalbumin on 7th, 14th, and 21st day. Treatment groups were given spirulina powder (500 mg/kg body weight) and salbutamol (1 mg/kg), respectively, after the induction of asthma. All three asthmatic groups were also exposed to cigarette smoke daily along with respective treatment for 4 weeks. Asthma induction caused an increase in total cell count in bronchioalveolar fluid (BALF), while spirulina treatment reduced total cells in BALF by 33.50% and salbutamol by 41.7%. Level of interleukins (IL) like IL‐4 decreased by 33.32% & 48.56% in G_1_ and G_2_. Similarly, IL‐5 and IL‐13 levels reduced by 40.9% & 49.9% and 18.62% & 38.02%, respectively, in G_1_ and G_2_. Serum levels of Immunoglobin‐E (Ig‐E) declined by 29.70% and 52.82%, while histamine levels were 26.23% & 45.58% less at the end of study in comparison to positive control. Moreover, histological analysis of lung tissue revealed that both spirulina and salbutamol effectively reduced ovalbumin and cigarette smoke‐induced moderate to severe necrosis, architectural changes, and congestion. It was concluded that salbutamol showed better results however, spirulina also effectively reduced mild to moderate allergic symptoms in dose‐dependent manner. Nutraceutical and functional foods are considered helpful in mitigating oxidative stress‐mediated health problems. Spirulina has its unique nutritional profile including phycobiliproteins, phytochemicals, and antioxidant vitamins which make it useful against several ailments. Considering its antioxidant role, current study was focused on exploring therapeutic efficacy of spirulina against stress biomarkers in asthmatic model. Outcomes of present research also demonstrated beneficial effect of spirulina in modulating allergic symptoms. In this regard, ancient concept of “medicine food homology” can be implemented and spirulina can be incorporated in food for additional benefits. However, further research regarding safety aspects is needed for its use in clinical practice for humans.

## Graphical abstract

Ovalbumin and cigarette smoke successfully developed asthma in treatment groups, while spirulina effectively reduced all asthma‐specific stress biomarkers used in the study. Furthermore, spirulina and salbutamol both reduced moderate to severe necrosis, architectural changes, and congestion.

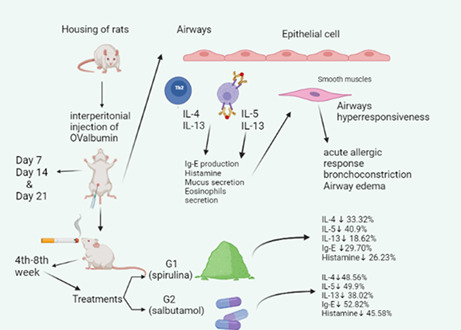

## 1 INTRODUCTION

According to the traditional definition, an allergy is a set of adverse effects that are triggered by the secondary immune response while interacting a foreign antigen which is generally harmless (Dhudasia et al., 2021). Allergies are complex disorders associated with various factors including genetic and environmental, etc. Their association can be determined by evaluating the expression of disease and its various phenotypes (Pawankar et al., 2011). Allergic disorders are prevalent among all age groups and are common worldwide resulting a major health burden. In this context, various mechanisms are involved but immunoglobin‐E (Ig‐E)‐mediated reactions are most prominent. Asthma, food allergy disorders, rhinitis, gastrointestinal disorders, and atopic dermatitis are categorized as major chronic allergic disorders that may cause irreversible impairments to effected organ (Dhudasia et al., 2021). Asthmatic response of lung to the inhaled allergen is often described by two different phases, that is, early‐phase response (EPR) and late‐phase response (LPR). In EPR, allergic symptoms occur within 1 h of exposure to allergens whereas in LPR, symptoms occur several hours later with prolonged persistence and severity (Drazdauskaitė et al., 2021; Gandhi et al., 2017). Pathophysiological mechanism of asthma involves oxidative stress and increased exposure to reactive oxygen species (ROS) (Czerska et al., 2016). ROS are produced extrinsically by environmental factors, that is, smoking and air pollutants and internally by metabolic reactions (Wang et al., 2021). These overproduced ROS are balanced by endogenous antioxidant defense system involving enzymatic antioxidants like SOD, CAT Gpx, etc., while non‐enzymatic antioxidants include vitamin C, E, and zinc, etc. (Mittal et al., 2014; Ramzan et al., 2021). Studies have reported significant association of these oxidative stress biomarkers with the characterization of asthma, especially in adults (Czerska et al., 2016; Ramzan et al., 2021). Interaction with allergens (animal fur, pollens, molds, dust mites, and cockroaches) and non‐allergens (exercise, smoke, cold air, and infections) causes differentiation of T helper cell in T cell type‐2 which further cause cascade of allergic responses. Hence, in response to an increase in Th2 levels, cells release inflammatory cytokines like interleukins (IL‐4, IL‐5, IL‐10, and IL‐13). These cytokines promote inflammation by initiating eosinophilic response resulting in an increased Ig‐E production which binds to receptors on mast cells and initiates the release of inflammatory mediators like histamine and leukotrienes (Bulowa et al., 2018; Fleischer et al., 2021).

In the United States, the use of natural products, dietary supplements, and vitamins as an additional treatment shows 40% of the conventional therapies (Costa et al., 2010; Ventola, 2010). According to various studies, alternative medications associated with natural products by involving multiple immunomodulatory biochemical pathways could reduce the pathogenesis of most diseases (Jiang et al., 2021; Panossian et al., 2021; Shaw et al., 2021; Xing et al., 2021). A step‐by‐step approach is required to manage allergic diseases. Shared care plan and self‐management lead to improvement in asthma outcomes (Wilson et al., 2010). Furthermore, knowledge regarding proper inhaler strategies, avoidance from irritants & allergens, and medication compliance are important for each patient suffering from asthma.

For the last 5000 years, products derived from plants have been useful for the treatment of asthma known as traditional or folk medicines against asthma. Plants have many secondary metabolites that possess bronchodilator and anti‐inflammatory properties, thereby enhancing anti‐asthmatic activities. Terpenoids, flavonoids, and phenolic acids are the main constituents that increase anti‐asthmatic properties. Nowadays, people are more interested in healthy foods for disease prevention which contain high nutritional value and play a vital role in promoting human health (Dawoud, 2016). Beneficial microalgae have been a part of human diet and animal feed since decades. It grows in fresh and marine water as filamentous organism. Notably, a significant amount of nutrients is present in these microorganisms including essential amino acids and fatty acids along with many other bioactive components (Abdulmumin, 2013). Spirulina is a spiral‐shaped threadlike alga that belongs to family Oscillatoriaceae. It is naturally found in pollution‐free, mineral‐rich, alkaline, and high pH water. It flourishes in alkaline water lakes where it is not possible for other micro‐organisms to survive (Michael et al., 2019). Spirulina acquires its blue‐green color due to the variety of pigments having their functional properties like chlorophyll a, β‐carotene, zeaxanthin, and phycobiliproteins (C‐phycocyanin, A‐phycocyanin, and phycoerythrin) which acts as a free radical scavenger and reduces inflammation by correcting function of antioxidant enzymes present in the body (Arashiro et al., 2020). It has gained much attention as nutraceutical for its immunity‐boosting effects (Mathur, 2019). Even though spirulina comprises good nutritional composition, its use and cultivation in Pakistan are very limited. Several researchers have demonstrated its antioxidant and anti‐inflammatory potential (Asghari et al., 2016; Farag et al., 2016; Lechuga Morente, 2021; Masuda & Chitundu, 2019; Simon et al., 2018; Soltani et al., 2012) however, studies regarding its antiallergic effects are limited. Therefore, the present study was designed with the aim to check antiallergy and immunomodulatory effects of spirulina with special references to asthma.

### 1.1 Hypothesis

Spirulina powder may play an effective role in attenuating asthma‐specific oxidative stress biomarkers.

## 2 MATERIAL AND METHODOLOGY

### 2.1 Bioevaluation Trials

For bio‐efficacy study, 40 female *Sprague–Dawley* rats of weight 200–250 g were procured and kept at the Animal Room of National Institute of Food Science and Technology, University of Agriculture, Faisalabad. This study was conducted following the guidelines of Pakistan Biosafety Committee 2005, Punjab Biosafety Rules 2014, and Bioethics Protocols. Rats were kept in glass boxes 1 week prior to the experimental trial for adaptation under constant care, with food and water at 23–25°C. After 1 week, the rats were randomly divided into four treatment groups (*n* = 10) to check antiallergic potential of spirulina powder. Purposely, the G_0–I_ was fed on normal diet and served as negative control whilst G_0–II_ was dependent on normal diet along with induction of asthma to serve as positive control. Whereas G_I_ and G_2_ were given spirulina powder (500 mg/kg body weight) and salbutamol (1 mg/kg), respectively after the induction of asthma. Later three groups were also given cigarette smoke daily during the treatment period in Chamber (25 cm high, 40 cm wide, and 30 cm long). Two cigarettes were placed in a small open hole at one side of glass chamber. This set‐up produced side‐stream smoke that provoked the passive smoke or environmental tobacco smoke exposure. Trial was carried out for 7 weeks, first 3 weeks for asthma induction and next 4 weeks for treatment. After the set time, overnight fasted rats were decapitated, blood, serum, and tissue samples were collected to check the efficacy of spirulina powder against oxidative stress biomarkers.

### 2.2 Induction of bronchial asthma

Asthma was induced in rats by giving them intraperitoneal injection of 1 mg ovalbumin and 20 mg aluminum hydroxide Al (OH)_3_ mixed in phosphate buffer saline (pH:7.4) on 7, 14, and 21st day. All three groups (except negative control group) were exposed to cigarette smoke (to enhance allergic symptoms related to asthma) on daily basis along with respective treatment for 4 weeks. (Abdеlaziz et al., 2018; Ezz‐Eldin et al., 2020).

### 2.3 Experimental design for in vivo study (Table 1)

**TABLE 1:** Treatment plan for efficacy study

Groups	Treatments
G_0–I_ (Negative control)	Normal diet
G_0–II_ (Positive control)	Normal diet + Ovalbumin‐Induced Asthma (OIA) + Cigarette Smoke (CS)
G_1_	Normal diet + OIA + CS + spirulina powder
G_2_	Normal diet + OIA + CS + salbutamol

### 2.4 Blood sampling and analysis

After the completion of trial, rats were anesthetized and sacrificed. Blood and serum samples were collected in different tubes considering analyses to be performed.

### 2.5 Hematology

The effect of spirulina powder on oxidative damage of cells caused by allergen and cigarette smoke was assessed by performing white blood cells count (Qinghua et al., 2016). Purposely, a hematology analyzer and Soluplastin‐kit (Wiener‐Lab, Argentina) were used. For serum collection, coagulated blood samples were centrifuged at 4000 rpm for 15 min. The upper serum layer was cautiously collected and kept at −40°C for analyses (Ezz‐Eldin et al., 2020).

### 2.6 Detection of asthma‐specific serum oxidative stress biomarkers

The concentrations of Ig‐E, histamine, and cytokines (IL‐4, 5, and 13) in serum were detected using ELISA kits. The samples were examined on an automated ELISA plate reader (Arora et al., 2016; Hirano et al., 1989; Martin et al., 2006).

### 2.7 Bronchoalveolar lavage fluid (BALF) collection

Bronchoalveolar lavage is a useful method for the identification of pathologies associated with lungs. Bronchoalveolar lavage(BAL) is a clinical practice in which Fluid is squirted into the lung and then recollected for WBC estimation. High amount of white blood cells present in lungs indicates the severity of lungs inflammation. Purposely, trachea and lungs were exposed by dissection of the chest cavity. For BALF collection, PE‐90 polyethylene tube linked to a needle hub was gradually introduced into the trachea and 5 ml of cold phosphate‐buffered saline (PBS) was injected. The PBS was allowed to stay in the lungs for 30 s, while the thoracic area was slightly massaged and suctioned gradually through the needle hub. This procedure was done three times in all groups and BALF was collected followed by centrifugation of fluid for 10 min. Afterward, BAL cells were suspended in 1 ml of saline, stained with Turk's solution, and calculated by a hemocytometer (Song et al., 2010).

### 2.8 Histopathological examination

Histological examination of lungs and liver tissues of rats was performed by following the protocols of Kafle et al. *(* 2018). At the end of the bio‐efficacy trial, lungs and liver of rats were removed followed by fixation in 10% formalin in saline. After 72 h of fixation, the sections were cut and embedded in paraffin wax for dehydration. After cooling, the paraffin blocks were decalcified in water for 40–50 min followed by washing and kept frozen till trimmed. Five‐micrometer‐thick pieces were taken and stained with hematoxylin and eosin on glass slide to assess degree of inflammation. The stained slides were observed under light microscope for digital image collection.

### 2.9 Statistical analysis

The obtained data from each parameter were subjected to statistical analysis to check the level of significance. Completely Randomized Design was applied by using Statistix 8.1 software. Furthermore, Tukey's HSD test was applied to analyze the differences in means (Montgomery, 2008).

## 3 RESULTS

### 3.1 Physical signs of allergy and asthma progression

After first injection of ovalbumin, no prominent changes were observed in the behavior of rats. No significant activity loss, hair loss, or irritation in nose were observed. On the 21st day of study (last ovalbumin injection), a gradual loss of activity was recorded. Rats of negative control group were active, while reduced activity was noticed in the later three groups. Moreover, the forced breathing rate was higher in asthmatic rats (G_0–II_, G_1_, and G_2_) than negative control (G_0–I_) alongside an increase in fluid intake. Non‐substantial hair loss was also observed in some rats. After 1 week of last ovalbumin injection, rats were given respective treatments along with cigarette smoke daily. During smoke exposure, mostly rats were calm at initiation and subsequently start sleeping as exposure time increased. This behavior was noticed on daily basis. Within 1 week of treatment and cigarette smoke exposure, significant hair loss and decline in activity were observed in the asthmatic rats. An increase in fluid intake was also evident. All these indicators were increasing with every passing week in positive control (G_0–II_). Symptoms were also present in G_I_ (spirulina treatment) and G_II_ (drug treatment) however, level of severity was less as compared to G_0–II_ (Table 2).

**TABLE 2:** Physical signs of allergic asthma progression in experimental rats

Treatment	Hair Loss	Nose Scratch	>Forced Breathing	<Activity	>Irritant Behavior	>Fluid Intake
G_0–I_	−	−	−	−	−	−
G_0–II_	++	++	++	++	++	++
G_1_	++	+	+	+	+	++
G_2_	++	+	+	+	+	+

Abbreviations: CS, Cigarette Smoke; OIA, Ovalbumin‐Induced Asthma. G_0–I_ (Negative control), normal diet; G_0–II_ (Positive control), Normal diet + OIA + CS; G_1_, Normal diet + OIA+ CS + spirulina powder (500 mg/kg body weight); G_2_, Normal diet + OIA+ CS + salbutamol (1 mg/ kg). *Note*: (−) = Not Present, (+) = Mildly Present, (++) = Significantly Present.

### 3.2 Leukocyte's indices

Results of the present study inferred an increase in total WBCs and eosinophils and a reduction in serum lymphocyte count of asthmatic rats as compared to negative control. All these variations are in favor of asthma induction in animal model. Besides, all asthmatic rat groups exhibited significant (*p* < .05) differences in leukocytes indices. Treatment group fed on spirulina powder showed 22.75 and 18.42% reduction in total WBCs and eosinophils count, whereas 16.15% increase in lymphocytes was observed when compared to positive control. Likewise, the intake of salbutamol reduced total WBCs and eosinophils by 24.86% and 21.05% and an increase in lymphocytes was 20.28%. Both spirulina and salbutamol were effective in reducing the severity of disease however, drug demonstrated better results compared to spirulina primarily due to the difference in effective doses (Table 3).

**TABLE 3:** Mean values for leukocyte indices of experimental rats

Experimental groups	White blood cells (10^3^/μl)	Lymphocytes (%)	Eosinophils (%)
G_0–I_	7.51 ± 0.30^c^	52.95 ± 4.35^a^	2.10 ± 0.25^c^
G_0–II_	18.92 ± 1.31^a^	42.73 ± 3.73^b^	3.82 ± 0.34^a^
G_1_	14.56 ± 0.53^b^	49.60 ± 8.23^a^	3.10 ± 0.33^b^
G_2_	14.23 ± 0.55^b^	51.36 ± 9.58^a^	3.01 ± 0.31^b^

Abbreviations: CS, Cigarette Smoke; OIA, Ovalbumin‐Induced Asthma. *Note*: Data values represent means ± SD (*n* = 10); alphabet a indicates highest mean value, while values sharing same alphabetical letter are statistically alike; G_0–I_ = (Negative control), G_0–II_ (Positive control) = Normal diet + OIA + CS, G_1_ = Normal diet + OIA + CS + spirulina powder (500 mg/kg body weight), G_2_ = Normal diet + OIA+ CS + salbutamol (1 mg/kg).

### 3.3 Asthma‐specific oxidative stress biomarkers

Means regarding asthma‐specific oxidative stress biomarkers in serum (histamine, Ig‐E, IL4, 5, and 13) of all treatment groups are expressed in Table 4. Furthermore, all asthmatic rat groups exhibited significant (*p* < .05) differences in asthma‐specific oxidative stress biomarkers. Levels of stress biomarkers conspicuously varied among normal and asthmatic rats as these are released with the onset of disease or allergic conditions. Therefore, comparison of treatment groups was made with a positive control to assess the extent to which treatments (spirulina and salbutamol) affected disease conditions. Levels of IL‐4 decreased by 33.32 and 48.56% in G_1_ and G_2_, likewise, IL‐5 & IL‐13 by 40.90% & 49.90% and 18.62% & 38.02% in both groups, respectively. Serum levels of Ig‐E in both groups exhibited decline of 29.70% and 52.82%, while histamine reduced to 26.23% and 45.58% on trial completion.

**TABLE 4:** Asthma‐specific biomarkers of oxidative stress in experimental rats

Experimental Groups	IL‐4 (pg/ml)	1 L‐5 (pg/ml)	IL‐13 (pg/ml)	Ig‐E (IU/ml)	Histamine (ng/ml)
G_0–I_	28.53 ± 3.47^d^	19.64 ± 2.46^d^	10.77 ± 0.99^d^	31.51 ± 2.90^d^	15.40 ± 1.42^d^
G_0–II_	183.80 ± 16.94^a^	175.05 ± 16.13^a^	65.83 ± 6.07^a^	205.61 ± 33.06^a^	128.30 ± 12.98^a^
G_1_	122.54 ± 11.29^b^	103.28 ± 9.52^b^	53.57 ± 4.94^b^	144.42 ± 13.31^b^	94.64 ± 7.19^b^
G_2_	94.53 ± 8.71^c^	87.53 ± 8.06^c^	40.80 ± 4.64^c^	96.99 ± 4.38^c^	69.82 ± 6.52^c^

Abbreviations: CS, Cigarette Smoke; OIA, Ovalbumin‐Induced Asthma. *Note*: Data values represent means ± SD (*N* = 10); alphabet a indicates highest mean value, while values sharing same alphabetical letter are statistically alike; G_0–I_ = (Negative control), G_0–II_ (Positive control) = Normal diet + OIA + CS, G_1_ = Normal diet + OIA + CS + spirulina powder (500 mg/kg body weight), G_2_ = Normal diet + OIA+ CS + salbutamol (1 mg/kg).

### 3.4 Total cell counts in BALF

**FIGURE 1:**
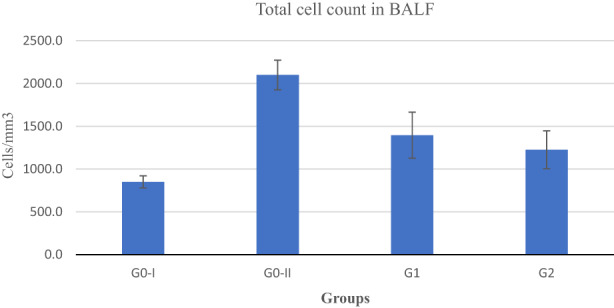
Total cell count in BALF of experimental rats. Negative control rats (_G0–I_), Positive control rats (G_0–II_), Spirulina‐treated rats (G_1_), Salbutamol‐treated rats (G_2_)

Mean values pertaining to total cell count in the bronchoalveolar fluid (BALF) are displayed in Figure 1. The analysis depicted momentous differences among the groups of asthmatic rats. Findings revealed that both treatments (spirulina and salbutamol) markedly reduced cell infiltration in lungs. Moreover, comparison with positive control revealed that spirulina treatment reduced total cells in BALF by 33.52%, while salbutamol 41.66%.

### 3.5 Histological examination of the lung in normal and asthmatic rats

**FIGURE 2:**
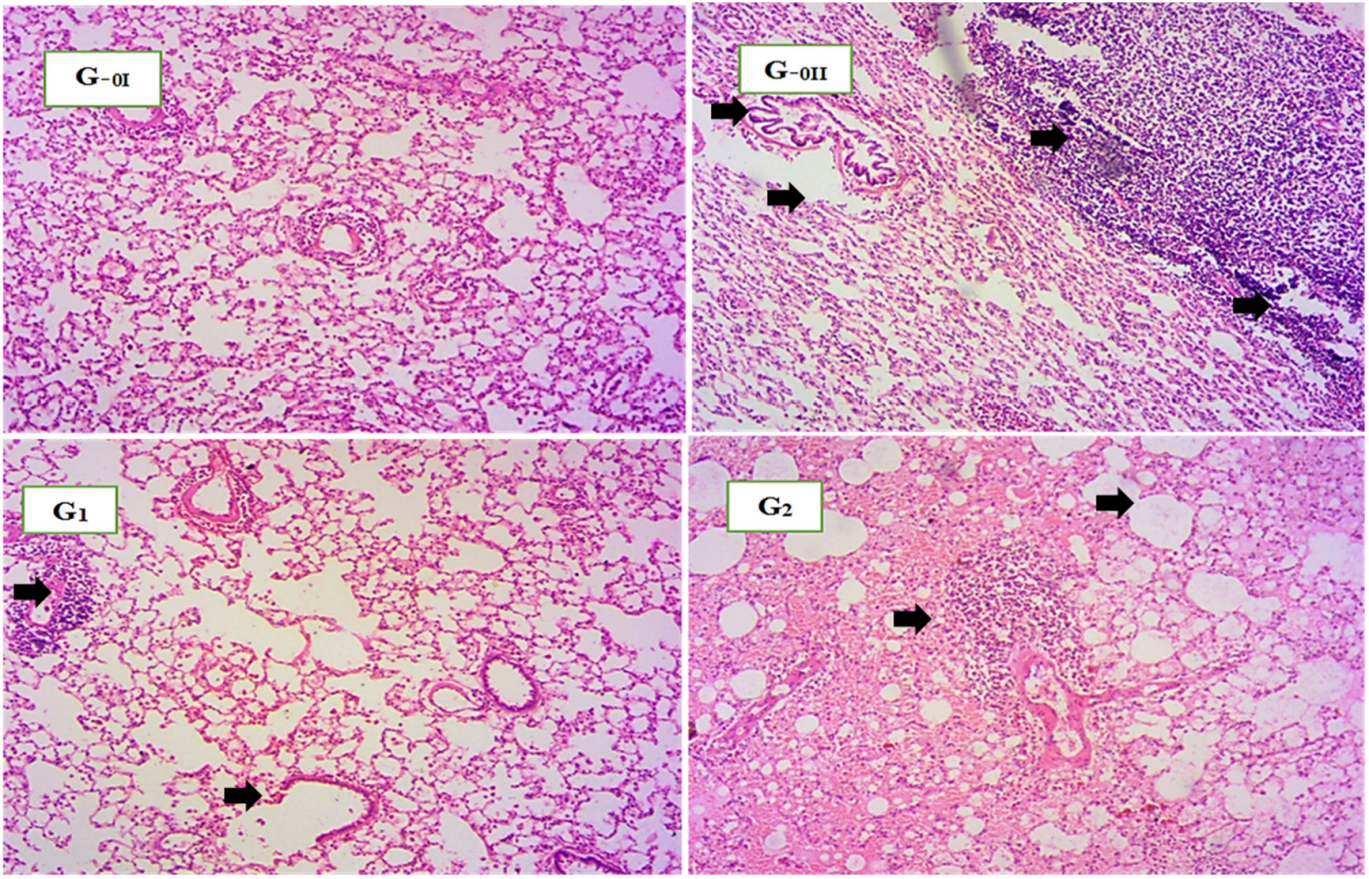
Histopathological study of pulmonary parenchyma. Negative control rats (G_0–I_), Positive control rats (G_0–II_), Spirulina‐treated rats (G_I_), Salbutamol‐treated rats (G_2_)

Histological examination of lung tissue of negative control revealed that no noticeable changes were observed in appearance of pulmonary parenchyma. Furthermore, alveolar spaces were normal, and no cell infiltration was noticed. Whereas lungs tissue of ovalbumin‐sensitized rat groups (G_0–II_, G_1_, and G_2_) demonstrated clear bronchial infiltration of leukocytes. Pulmonary damage, interstitial edema, and increased alveolar spaces were also detected in many regions. Arrows (Figure 2) are indicating infiltration of leukocytes edema and increased alveolar spaces Exposure to cigarette smoke along with asthma induction severely induced imbalance between oxidants and antioxidant enzymes in lungs. In positive control, lung damage was severe in comparison to treatment groups. Both spirulina and salbutamol efficiently mitigated oxidative damage in lungs caused by asthma and cigarette smoke. In salbutamol‐treated rats (G_2_), fewer inflammatory cells and mild degree of alveolar damage in some areas were observed. Moreover, normal architecture of pulmonary parenchyma was also preserved due to constant treatment. Similarly, treatment with spirulina (G_1_) also effectively prevented infiltration of leukocytes. Oxidative damage and injury of lung tissues were also declined. Histopathology results revealed that ovalbumin along with cigarette smoke caused cellular damage. Moreover, treatment with spirulina helped in preserving lung tissue from oxidative damage in dose‐dependent manner.

## 4 DISCUSSION

Natural products exhibit positive effects against inflammatory and allergic diseases. Alternative medications derived from natural sources can prevent multiple diseases by various immunomodulatory biochemical pathways. Since the spirulina is an excellent source of protein, numerous researchers have studied its role in overcoming malnutrition however, its effect on allergic diseases needs to be explored further. Therefore, the present investigation was focused on the use of spirulina against asthma based on its therapeutic properties. It is a well‐known fact that asthma leads to chronic breathing problems and prolonged effects can cause permanent changes at molecular level. Animal studies provide an insight and understanding of possible underlying mechanism of disease. Furthermore, tobacco smoke comprises toxins (nicotine, carbon monoxide, ammonia, and acetone), chemically reactive compounds, and oxidants. These toxic compounds can directly affect pulmonary functioning revealing pro‐inflammatory, mutagenic, and carcinogenic properties. Inhalation of oxidants causes direct lung injury and initiation of inflammatory reactions leading to tissue damage (Stabile et al., 2021). Consequently, oxidative stress plays a major role in pathogenesis of several inflammatory lung disorders such as asthma and chronic obstructive pulmonary disease (COPD) (Strzelak et al., 2018).

Physical signs and symptoms are the first parameters to assess whether allergic symptoms are developed. In this regard observations of current research are in accord to other investigational studies. Earlier, a group of researchers conducted human trial to check the effects of spirulina intervention on oxidative stress, antioxidant status, and lipid profile between COPD patients and controls. Among smokers and non‐smoker category, smokers experienced more symptoms like difficulty in breathing and wheezing. According to many patients, smoking was also linked to less food intake and reduced weight. While patients taking spirulina with medication observed better working capability and appetite. Moreover, weight gain was also improved and patients experienced less flare‐ups and breathing difficulty sessions (Ismail et al., 2015). Similarly, Alazemi et al. (2018) conducted a study to assess the severity of asthma in smokers and non‐smokers. Smokers exhibited worse signs of wheezing and shortness of breath related to non‐smoker asthmatics. Both groups showed similar spirometry; however, the use of standard drug (salbutamol) was more frequent in smoker asthmatics. In the present study, nasal scratch and wheezing (difficulty breathing) were observed in all three groups given ovalbumin injections in comparison to control group. After smoke exposure for 4 weeks, symptoms were higher along with significant wheezing. Ovalbumin‐induced allergy caused prominent symptoms, while cigarette smoke enhanced chest tightening and wheezing. On completion of treatment period, considerable reduction in all allergic symptoms was observed in both treatment groups (spirulina and salbutamol). Earlier, another group of peers developed animal model of asthma with ovalbumin sensitization and exposure to cigarette smoke. Their results revealed that exposure of cigarette smoke to fetus in uterus and newborn substantially reduced the lung functions. It has also been noticed that cigarette smoke exposure caused wheezing, coughing, sputum production, airway hyperresponsiveness, and decreased forced expiratory volume (Barrett et al., 2002). Numerous studies have reported similar results in terms of breathing and activity level (Chen et al., 2019; Sakat et al., 2018; Thakur et al., 2019).

Su et al. (2020) reported several physical changes in mice after sensitization and exposure of rats to cigarette smoke for successive 3 weeks. Ovalbumin‐induced allergy‐like symptoms and reduced pulmonary functions, while cigarette smoke enhanced the severity of these markers. They also indicated that with or without cigarette smoke, the decline in pulmonary function was noticed. Overall activity level of rats was reduced. Their findings denoted that cigarette smoke exposure caused reduction in feed intake while water intake was elevated. Rats behaved more irritably and their fur color turned yellow in comparison to control. Conclusively, all these studies agree with current experiment in terms of physical signs of disease implying that allergic asthma‐like symptoms were developed and bioactive agents of food helped reducing the severity of condition.

Asthma causes airway obstruction, characterized by infiltration of white blood cells, eosinophils & lymphocytes in lungs, mucus hypersecretion and hyperresponsiveness of airways to inhaled antigen (Zou et al., 2021). Many studies have reported an association of increased eosinophils level with severity of disease and elevated production of oxidants in asthmatic patients as compared to normal subjects (Bishopp et al., 2017; Chamitava et al., 2020; Parulekar et al., 2016). As a result, airway inflammation increases by activating different proinflammatory cells including T lymphocytes and eosinophils (Palmo et al., 2021) which play a vivid role in pathogenesis of disease by differentiation of T cells to Th2 cells (secreting IL‐4, IL‐5, and IL‐13) involved in humoral immunity against extracellular infections (Shakeri & Boskabady, 2017). Thus, the rise in the eosinophils level is a major feature of allergic asthma leading to many inflammatory responses (Nair & Prabhavalkar, 2021). Results of the present research are in harmony with previous trials which induced asthma in animal model and studied the effects of different treatments. Recently, Nair and Prabhavalkar (2021) developed asthma with ovalbumin and explored effect of curcumin and salbutamol on biomarkers of asthma and significant changes in hematological parameters of asthmatic rats were noticed in comparison to control. Furthermore, curcumin and salbutamol in combination effectively alleviated inflammation though levels of leukocytes were still high compared to normal rats. Another group of researchers, Thakur et al. (2019) developed an experimental model of asthma in rats using ovalbumin. Total leukocyte counts in the blood were markedly increased by 114% in the ovalbumin‐sensitized groups compared to normal rats. Similarly, eosinophils levels were significantly increased three times in the blood of asthmatic rats. In context to antioxidant effects of spirulina, Xiong et al. (2018) conducted an experiment to evaluate pharmacologic outcomes of ethanolic extracts of spirulina combined with fish oil (CFS) to mitigate asthma induced by ovalbumin in mice. Asthmatic mice were treated with dexamethasone (corticosteroid) and 0.41, 0.65, and 0.18 g/kg of CFS for 3 months. Their findings demonstrated that CFS reduced inflammatory cells count in serum and lungs. Total WBCs were decreased in all treatment groups as compared to positive control. The highest mitigation properties were observed in group receiving dexamethasone. After drug, the most effective treatment was 0.18 g/kg CFS in reducing WBCs count. The basic trend of their study in reducing severity of disease is in accordance with present outcomes. Similar results were displayed by some other researchers in this regard (Arora et al., 2016; Fitry et al., 2020). Earlier, Barret and his co‐workers (2002) developed allergic symptoms with inhalation of cigarette smoke and ovalbumin injection. According to their findings, total cells in BALF of normal rats were recorded as 3.48 ± 0.79 Cells × 10^5^/ml which increased to 5.33 ± 2.46 Cells × 10^5^/ml attributed to allergy progression. Infiltration of cells in lungs was 53.16% higher compared to normal rats. In reference to effect of drugs, the trend of present outcomes is in accordance with recent findings of two researchers Nair and Prabhavalkar (2021). They reported that salbutamol and dexamethasone (0.5 and 0.1 mg/kg) effectively reduced total and differential cell count in BALF of treatment groups. Total cell counts decremented 70% with combined drug treatment. Furthermore, saffron protected oxidative stress along with salbutamol by reducing 45% cell infiltration in lungs. Similarly, another group of peers reported the same effects of asthma induction. Ovalbumin‐sensitized rats showed a marked incline in total and differential cell count in BALF. Eosinophils, macrophages, and neutrophils numbers reduced substantially in treatment groups receiving (42.5 mg/kg) fruit extract. Total cell count increased from 7.00 ± 0.52 × 10^5^ to 11.17 ± 0.87 × 10^5^ cells/ml attributed to asthma induction, whereas antioxidants components of fruit extract lessened total cell count to 8.11 ± 0.45 and dexamethasone (drug) to 4.83 ± 0.48 × 10^5^ cells/mL (Arora et al., 2016). Likewise, outcomes of Thakur et al. (2019) revealed total cell count in BALF of normal and asthmatic rats 9 × 10^3^ cells/mm^3^ and 15 × 10^3^ cells/mm^3^, respectively. Cell count in BALF elevated up to 66.6% by asthma induction. Previously, Gholamnezhad et al. (2014) assessed effect of Fluticasone (corticosteroid) and Salmeterol (β2‐agonist) on total WBC count in BALF. Rats were administered both drugs during and after sensitization period. Normal cell level in BALF was 500, while Control and treatment groups showed 3000, 1000, and 1500 cell count/ml increment in BALF. Results demonstrated 66.60% and 50% reduction in total cell count due to treatment. Allergy induction increased overall ce0ll count in BALF up to 50–70% however, treatment with drugs showed a decline of 45%–70%. Research findings also indicated that treatment of allergic disorders with antioxidant‐rich plant foods have also displayed prophylactic properties as proclaimed by current exploration.

Generally, autophagy is conducive to cells' existence, keeping the homeostasis by eliminating necrotic organs and proteins in response to environmental stress inducers like hypoxia, temperature fixation, and infectious pathogens. Oxidative stress causes excessive autophagy that can cause adverse effects like cell death. It is an underlying factor in pathogenesis of various diseases, the pathological mechanism of allergic asthma starts with the differentiation of T cells to allergen‐specific T helper 2 cells, followed by the production of immunoglobulin E (Ig‐E) (Wang et al., 2021). It is an allergen‐specific immunoglobulin that attaches to its receptors (Fc receptors) and activates Ig‐E‐dependent mast cells which further release inflammatory factors like histamine and leukotrienes causing acute allergic reactions. On the other hand, Th‐2 helper and B cells (humoral immunity) also release interleukins (IL‐4, 5 & 13) which also infiltrate in lungs tissue and cause obstruction of airways and permanent remodeling if persist over the time. Eventually, the continuous triggering of interleukins by antigens causes chronic allergic asthma symptoms (Vo et al., 2012).

Earlier findings have also shown that cigarette smoke could exacerbate ovalbumin‐induced asthma and markedly enhance the expression of IL‐4 and IL‐13 genes (Sun et al., 2021). Glucocorticoids and β2‐agonists are mainly used in modern medicine to treat allergic asthma. However, long‐term use of glucocorticoids is prone to resistance (Wang et al., 2021). Besides, several marine algae have demonstrated role in blocking binding of Ig‐E to its receptors on mast cells and release of inflammatory factors like interleukins and histamine (Sugiura et al., 2008). Previously, Mao and his co‐researchers conducted a trial in 2005 to evaluate effects of spirulina supplementation on cytokines production in allergic patients. Allergic individuals were regularly administered 1 or 2 g spirulina for 12 weeks. They found that 2 g/day supplementation of spirulina considerably lowered IL‐4 levels by 32%. Likewise, Arora et al. (2016) illustrated similar pattern of increment in all cytokines in ovalbumin‐sensitized rats compared to normal. In the groups treated with fruit extract (31 and 42.5 mg/kg), IL‐4 levels were reduced by 17% and 24%, while IL‐5 levels by 17.1% and 28.2% in both interleukins. Nevertheless, group treated with dexamethasone exhibited reduction of 56.6% and 47.7%, respectively. Drug also alleviated Ig‐E levels by 58% and histamine 66.6%. Doses of fruit extracts showed reduction in histamine level as 25% and 41.60%. Recent findings also validate the previous outcomes of Thakur et al. (2019). The team of researchers worked on experimental model of asthma. Asthma induction method was comparable to current study with slight variations. They also showed increment in stress biomarkers in positive control. Levels of all three interleukins (IL‐4, IL‐5, and IL‐13) were in the range of 18–22, 17–28, and 130–160 ng/mL in asthmatic rats, which were 125, 77, and 220% higher than normal, respectively.

Nair and Prabhavalkar (2021) elaborated the effects of combined saffron extract and salbutamol on ovalbumin‐induced asthmatic rats. Different doses of extracts (30 & 60 mg/kg) with drug (0.5 mg/kg) were administered to animals for 28 days. Their results depicted positive effects of treatment combination in reducing stress and improvement of histopathological parameters. Levels of inflammatory cytokines incremented markedly in positive control in comparison to normal rats. Results demonstrated momentous decrease in IL‐4 (20% and 15%) and IL‐13 (60% and 55%) levels with the combination treatment. Similarly, another group of peers Chen et al. (2019) investigated protective effects of polyphenols in ovalbumin sensitized animal model. They deduced that allergic symptoms can be prevented by polyphenols in a dose‐dependent manner. Serum Ig‐E levels raised in positive control group from 10 to 40 ng/ml, while baicalin (polyphenol) reduced Ig‐E levels to 16 ng/ml. Similarly, histamine levels were also elevated from 1 to 4 ng/mL and reduced to 2.2 ng/ml by 200 mg/kg dose of baicalin. Serum IL‐4 levels also lowered to 28 ng/mL in comparison to positive control (55 ng/ml). Their results indicated 60, 45, and 49% reduction in Ig‐E, histamine, and IL‐4 levels, respectively. The findings of instant study are in line with the inferences of previous researches that demonstrated prophylactic role of plant‐based foods mainly attributed to their bioactive components.

The outcomes of histological examination in the present research are in accordance with the previous study of El‐Sokkary et al. (2007) who indicated that nicotine administration to rats substantially caused tissue damage and architectural changes which include edema and high infiltration of cells. Likewise, another group of researchers Cardoso et al. (2021) exposed rats to cigarette smoke for 60 days and proclaimed that daily exposure to cigarette smoke caused damage to lungs. Histological analysis of lung parenchyma showed noticeable increase in alveolar spaces and destruction of alveolar region compared to normal group. Moreover, treatment with Diallyl disulfide (bioactive component of garlic) preserved normal architecture of lungs, alveolar septa was intact and alveoli were normal in size. Comparable to current research, Zou et al. (2021) also developed ovalbumin‐induced asthma model and revealed that asthmatic symptoms were positively achieved. They reported marked infiltration of inflammatory cells in bronchial regions of mice and treatment with gentiopicroside (phytochemical) in dose‐dependent manner significantly reduced infiltration of cells. Furthermore, asthmatic mice also exhibited rise in goblet cells in alveolar region compared to normal mice. Regarding protective effective of spirulina against oxidative damage, current study is quite in harmony with Xiong and fellows (2018). They combined fish oil and spirulina extract for the treatment of airways inflammation and hyperresponsiveness in mice induced by ovalbumin. Normal control showed normal morphology of lungs, while ovalbumin sensitization markedly increased bronchial leukocytes infiltration. Interstitial edema and tissue damage were also observed. On the other hand, treatment with 0.8 and 1.6 g/kg spirulina & fish oil combination and dexamethasone (corticosteroid) helped reverse damage. Cell infiltration and edema was also reduced. Likewise, another investigation declared that treatment with R‐phycocyanin from algae for 48 h suppressed ovalbumin‐induced airway hyperresponsiveness. There were less infiltrated cells in bronchial region and R‐phycocyanin was able to reduce airway inflammation caused by allergens (Chang et al., 2011). In reference to antiallergic effects of drug, Nair and Prabhavalkar (2021) reported that no significant changes were observed in normal control. However, positive control group demonstrated mild eosinophil and moderate to severe leukocyte infiltration. In case of treatment groups, normal parenchyma of lungs was preserved and less infiltration of cells in bronchial region was reported.

## 5 CONCLUSION

Conclusively, ovalbumin sensitization initiated allergy‐like symptoms and cigarette smoke exposure played pivotal role in causing oxidative stress and enhancing allergic reactions. Both spirulina and salbutamol alleviated levels of oxidative stress biomarkers. However, salbutamol showed better results in mitigating asthma‐specific oxidative stress biomarkers, while spirulina is also effective in reducing overall oxidative stress caused by allergy and cigarette smoke in dose‐dependent manner primarily due to its bioactive components. The histological analysis of lung and liver tissues expounded that both spirulina and salbutamol effectively reduced ovalbumin and cigarette smoke‐induced moderate to severe necrosis, architectural changes, and congestion. Therefore, it is concluded that spirulina may have protective role against oxidative stress‐induced cellular damage.

## ACKNOWLEDGEMENTS

The authors are thankful to the Higher Education Commission, Pakistan, for providing funds under the project “Indigenous 5000 Fellowship Program.” The authors are also thankful to the Faculty of Food, Nutrition and Home Sciences, University of Agriculture, for providing the technical support.

## FUNDING INFORMATION

The work has been sponsored by the Higher Education Commission under the project “Indigenous 5000 Fellowship Program.”

## CONFLICT OF INTEREST

There is no conflict of interest.

## Data Availability

Data used to support the findings are included within the article.
